# Supplementary material for: STIM1-dependent peripheral coupling governs the contractility of vascular smooth muscle cells
Source: eLife. 2022 Feb 11;11:e70278. doi: 10.7554/eLife.70278 (PMC8947769; doi:10.7554/eLife.70278)
Supplement: Supplementary file 1. [file elife-70278-supp1.docx]

|  | **Control** | **Stim1-smKO** |
| --- | --- | --- |
| **BK:RyR2** | 2.6±0.25%, n=20 cells | 2.2±0.17, n=18 cells |
| **RyR2:BK** | 1.6±0.15%, n=20 cells | 1.8±0.18, n=18 cells |
|  |  |  |
| **TRPM4:IP3R** | 5.8±039%, n=15 cells | 5.2±0.46%, n=15 cells |
| **IP3R:TRPM4** | 5.1±0.44%, n=15 cells | 3.9±0.47%, n=15 cells |
|  |  |  |
