# Supplementary material for: STIM1-dependent peripheral coupling governs the contractility of vascular smooth muscle cells
Source: eLife. 2022 Feb 11;11:e70278. doi: 10.7554/eLife.70278 (PMC8947769; doi:10.7554/eLife.70278)
Supplement: Supplementary file 2. [file elife-70278-supp2.docx]

| Gene Name | Forward (5’ to 3’) | Reverse (5’ to 3’) |
| --- | --- | --- |
| *Kcnma1* | GCTTAAGCTCCTGATGATAGCC | AAGGTGGTTCCCAGGGTTAA |
| *Kcnmb1* | ACCAACAGTGCTCCTATATCCC | ACGCTGGTCTCGTTGACTTG |
| *Ryr2* | TGGAGGACATGCATCCAACA | TCCTATGCCTGACAAGAACTCC |
| *Trpm4* | TTCACGTACTCTGGCCGAAA | CGGGTAACGAGACTGTACACA |
| *Itpr1* | AACGTGGGCCACAACATCTA | CCAGGTTTCAGCATGGTTTGAA |
| *Itpr2* | CCTCAAGACAACCTGCTTCA | TGATGTGCTCCTCAAAGGAC |
| *Itpr3* | GCAGCGAGAAGCAGAAGAAA | GTTGTCAAACTTGTCCCTCTCC |
